# Association of serum uric acid with right cardiac chamber remodeling assessed by cardiovascular magnetic resonance feature tracking in patients with connective tissue disease

**DOI:** 10.3389/fendo.2024.1351197

**Published:** 2024-03-22

**Authors:** Yuanyuan Tang, Zhaoxia Yang, Jinyang Wen, Dazhong Tang, Yi Luo, Chunlin Xiang, Lu Huang, Liming Xia

**Affiliations:** Department of Radiology, Tongji Hospital, Tongji Medical College, Huazhong University of Science and Technology, Wuhan, Hubei, China

**Keywords:** connective tissue disease, serum uric acid, cardiovascular resonance magnetic imaging, feature tracking, right ventricular strain, right atrial strain

## Abstract

**Background:**

Right cardiac chamber remodeling is widespread in patients with connective tissue disease (CTD). Serum uric acid (SUA) is considered a potential independent risk factor for cardiovascular disease, and elevated SUA levels are often observed in patients with CTD. The correlation between SUA levels and right cardiac chamber remodeling remains unclear. This study investigated the association of SUA with right cardiac chamber remodeling as assessed by cardiac magnetic resonance feature-tracking (CMR-FT) in CTD patients.

**Methods and results:**

In this cross-sectional study, a total of 104 CTD patients and 52 age- and sex-matched controls were consecutively recruited. All individuals underwent CMR imaging, and their SUA levels were recorded. The patients were divided into three subgroups based on the tertiles of SUA level in the present study. CMR-FT was used to evaluate the right atrial (RA) longitudinal strain and strain rate parameters as well as right ventricular (RV) global systolic peak strain and strain rate in longitudinal and circumferential directions for each subject. Univariable and multivariable linear regression analyses were used to explore the association of SUA with RV and RA strain parameters. Compared with the controls, the CTD patients showed significantly higher SUA levels but a lower RV global circumferential strain (GCS) and RA phasic strain parameters (all *p* < 0.05), except the RA booster strain rate. RV GCS remained impaired even in CTD patients with preserved RV ejection fraction. Among subgroups, the patients in the third tertile had significantly impaired RV longitudinal strain (GLS), RV GCS, and RA reservoir and conduit strain compared with those in the first tertile (all *p* < 0.05). The SUA levels were negatively correlated with RV GLS and RV GCS as well as with RA reservoir and conduit strain and strain rates (the absolute values of *β* were 0.250 to 0.293, all *P* < 0.05). In the multivariable linear regression analysis, the SUA level was still an independent determinant of RA conduit strain (*β* = -0.212, *P* = 0.035) and RV GCS (*β* = 0.207, *P* = 0.019).

**Conclusion:**

SUA may be a potential risk factor of right cardiac chamber remodeling and is independently associated with impaired RA conduit strain and RV GCS in CTD patients.

## 1 Introduction

Connective tissue disease (CTD) is a heterogeneous group of chronic inflammatory diseases due to abnormal auto-immunity regulation and characterized by multiorgan involvement, including systemic sclerosis (SSc), systemic lupus erythematosus (SLE), primary Sjögren’s syndrome (pSS), idiopathic inflammatory myopathy (IIM), rheumatoid arthritis, and many other autoimmune diseases. Cardiovascular and pulmonary involvement is a common complication and one of the leading causes of death in patients with CTD. There are multiple clinical manifestations, presented as myocarditis, heart failure, valvular heart disease, coronary artery disease, pulmonary hypertension (PH) (1), and interstitial lung disease (ILD) (2). Right cardiac chamber remodeling, including chamber dilatation, wall hypertrophy, myocardial fibrosis, and cardiac insufficiency, plays an important role in the progression and prognosis of CTD. Both the primary involvement of the right ventricular (RV) myocardium and the secondary impact of ILD and PH on RV afterload can result in right cardiac chamber remodeling in patients with CTD. Echocardiography is limited by an acoustic window and poor reproducibility in assessing the right heart. Cardiac magnetic resonance (CMR) imaging is the current gold standard for quantitatively evaluating cardiac structure and function with high reproducibility. CMR feature tracking (CMR-FT) is a promising technique that evaluates myocardial deformation and provides additional information for subclinical cardiac dysfunction (3). Thus, RV and right atrial (RA) strain parameters may be more sensitive to assess the changes in right heart function and mechanics. Evidence supporting the early diagnostic and prognostic value of RV and RA strain parameters in CTD patients with cardiovascular involvement has accumulated over the years (4–6).

Serum uric acid (SUA) is the final metabolic product of purine degradation, which correlated with multiple cardiovascular risk factors and could be considered an independent predictor for several adverse cardiovascular outcomes (7). Previous studies demonstrated that an elevated SUA level was a potential risk factor of cardiovascular damage in patients with CTD (8, 9). Elevated SUA levels not only increase the risk of developing PH and serve as a marker for screening PH in CTD (10, 11) but may also be predictors of clinical prognosis in patients with CTD-associated pulmonary arterial hypertension (CTD-PAH) (12). Another study showed that SUA levels were correlated with RA pressures and RV function in patients with heart failure with preserved ejection fraction (13). However, the correlation between SUA levels and right cardiac chamber remodeling in patients with CTD remains unclear. Accordingly, the present study aimed to evaluate the right cardiac chamber remodeling in patients with CTD and to investigate the association of SUA with impaired RV and RA strain parameters as assessed by CMR-FT.

## 2 Materials and methods

### 2.1 Study design and population

**Figure 1:**
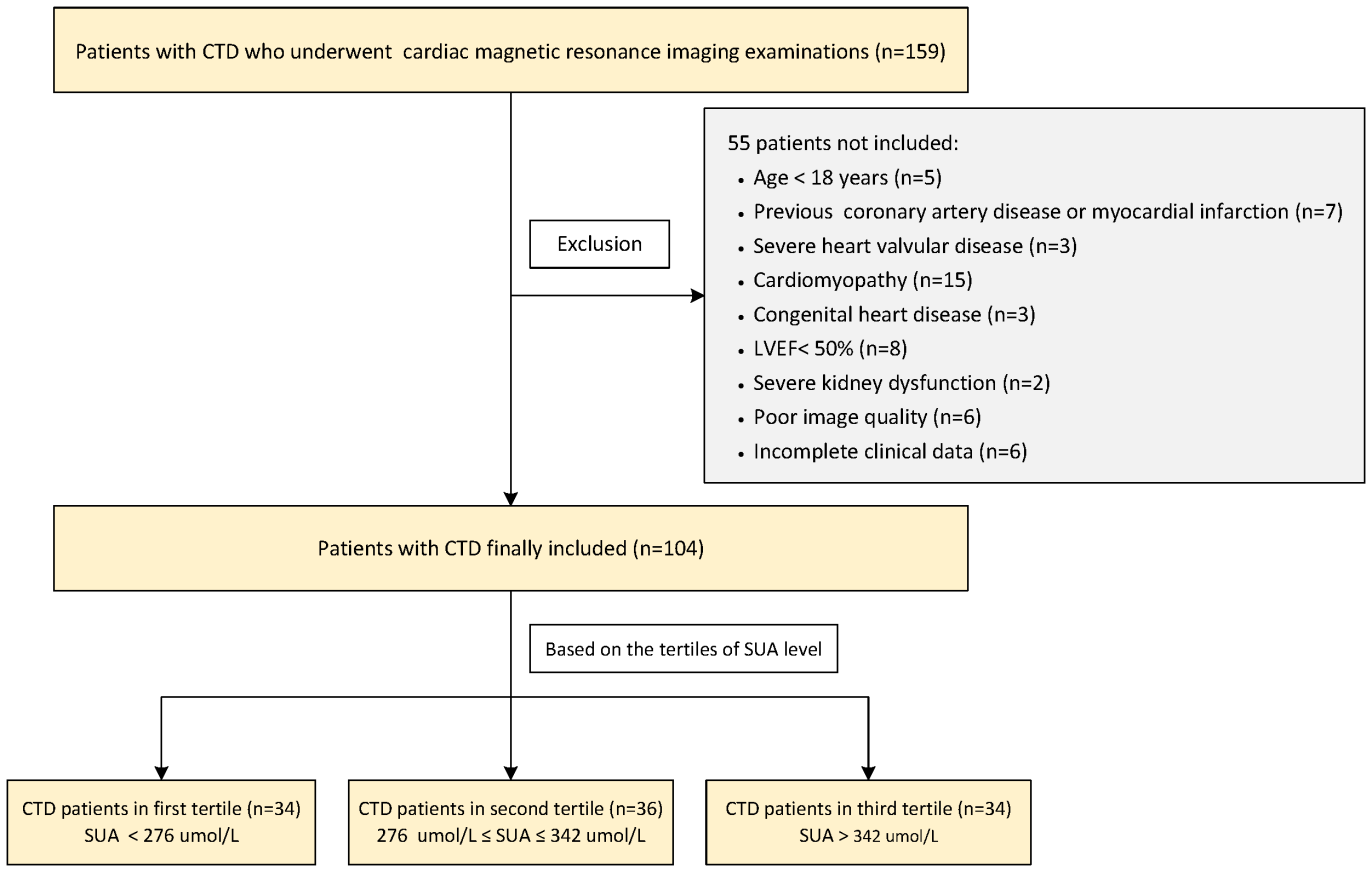
Flowchart of the CTD patients’ enrollment. CTD, connective tissue disease; LVEF, left ventricular ejection fraction; SUA, serum uric acid.

This cross-sectional study enrolled 159 consecutive patients with CTD who underwent 3.0T CMR examination from January 2015 to July 2023 in our hospital. The diagnosis of CTD was according to the American College of Rheumatology diagnostic criteria or the European League Against Rheumatism for IIM (14), SLE (15), SSc (16), pSS (17), and rheumatoid arthritis (18) as well as sharp criteria for mixed connective tissue disease (MCTD) (19). Overlap syndrome was defined as patients with two or more clinical features of CTD at the same time. Undifferentiated connective tissue disease (UCTD) was defined as patients with clinical and laboratory features suggestive of systemic autoimmune diseases but cannot be classified into any of the defined CTD classification criteria. The exclusion criteria were as follows: (1) age <18 years; (2) a history of coronary artery disease; (3) severe heart valvular disease, cardiomyopathy, and congenital heart disease; (4) left ventricular ejection fraction (LVEF) <50%; (5) severe kidney dysfunction (estimated glomerular filtration rate <30 ml/min/1.73 m^2^); (6) poor image quality; and (7) incomplete clinical data. Finally, a total of 104 CTD patients (comprising 49 patients with IIM, 29 with SLE, six with SSc, five with pSS, eight with overlap syndrome, two with rheumatoid arthritis, four with UCDT, and one with MCTD) were retrospectively included. The patients were divided into three subgroups based on the tertiles of SUA level in the present study. CTD patients with preserved RVEF (defined as RVEF ≥50%) were analyzed as a subgroup, too. Meanwhile, 52 age- and gender-matched individuals with no history of cardiovascular or systematic diseases were selected as the control group. This study protocol was approved by the Institutional Review Board of our hospital (TJ-IRB20230914), and the requirement for written informed consent was waived due to the retrospective design. The patients’ flowchart of this study is shown in **Figure 1**.

### 2.2 Clinical and laboratory data

Venous blood samples were obtained from a peripheral vein within 48 h of the CMR examination as well as without receiving any urate-lowering therapies. SUA, estimated glomerular filtration rate, total cholesterol, triglyceride, high-density lipoprotein-cholesterol, and low-density lipoprotein-cholesterol were measured in the laboratory department of our hospital. Pulmonary fibrosis was defined as reported on thoracic computed tomography imaging by specialist radiologists. PH was defined as systolic pulmonary arterial pressure >36 mmHg on echocardiography as measured by experienced doctors or mean pulmonary arterial pressure ≥20 mmHg on right-sided heart catheterization (20).

### 2.3 CMR scanning protocol

All patients and controls underwent a standard clinical protocol using 3.0T magnetic resonance imaging (MRI) scanners (MAGNETOM Skyra, Siemens Healthcare, Erlangen, Germany) with an 18-channel phased-array receive coil in the supine position. A steady-state free-precession sequence with electrocardiogram triggering and respiratory gating was used to assess cardiac structure and function. Short-axis cine images covering the right ventricle (RV) from apex to tricuspid annulus, as well as standard cine long-axis four-chamber view, were acquired. Typical imaging parameters were as follows: echo time/repetition time, 1.4/37.7 ms; flip angle, 55°; field of view, 360 × 360 mm^2^; acquisition matrix size, 192 × 146; slice thickness, 8 mm; 25 phases per cardiac cycle.

### 2.4 Cardiac function and feature tracking analysis

Post-processing of all CMR images was analyzed using an offline customized software CVI42 (version 5.13.9, Circle Cardiovascular Imaging Inc., Calgary, Canada). Right and left ventricular endocardial and epicardial contours were drawn manually at the end of diastole and systole in all short-axis cine images excluding the papillary muscles and trabeculae. Biventricular end-diastolic volume (EDV), end-systolic volume (ESV), stroke volume, myocardial mass, and ejection fraction (EF) were calculated. Right atrial (RA) volumes were measured in CVI42 function LAX biplanar module using the biplane area–length method on the cine CMR 4-chambers views, excluding the confluence of the superior and inferior vena cava, as well as the atrial appendage. RA maximum (*V*_max_) and minimum volumes (*V*_min_) were assessed at the end of ventricular systole and diastole, respectively. Pre-atrial volumes (*V*_pre-A_) were obtained before atrial contraction. The functions of RA were calculated by the following equations as previously described (21): (1) reservoir function: total emptying fraction (EF_t_) = (*V*_max_ − *V*_min_)/*V*_max_ × 100%; (2) conduit function: passive emptying fraction (EF_p_) = (*V*_max_ – *V*_pre-A_)/*V*_max_ × 100%; (3) booster pump function: active emptying fraction (EF_a_) = (*V*_pre-A_–*V*_min_)/*V*_pre-A_ × 100%. All volumes and RV myocardial masses were indexed to body surface area before analysis.

The myocardial deformation parameters of the RV free wall (RVFW) as well as the RA were evaluated in CVI42 feature-tracking module. The endocardial and epicardial contours of RV and RA were manually drawn using a point-and-click approach on the end-diastolic images, respectively. Subsequently, the contours were automatically tracked throughout the cardiac cycle while also checking the quality of the automatic tracking and adjusting the initial contour manually if necessary. RV global circumferential strain (GCS) and global circumferential strain rate (GCSR) were derived from tracking the RVFW in the four-chamber view and short-axial views, while the global longitudinal RV and RA strain and strain rates were quantified based on the four-chamber view only (**Supplementary Figures S1, S2**). RV radial strain parameters were not evaluated due to the high variability as previously described (22). Three aspects of RA phasic function were quantified for each subject as follows: (1) RA reservoir function (total strain [*ε*_s_] and strain rate [SRs]), (2) RA conduit function (passive strain [*ε*_e_] and strain rate [SRe]), and (3) RA booster function (active strain [*ε*_a_] and strain rate [SRa]). Above all, the results were based on the average of three independent repeated measurements.

### 2.5 Reproducibility of RV and RA strain analysis

The inter- and intra-observer variabilities for RV and RA strain and strain rate parameters were obtained from 40 randomly selected subjects (10 controls and 30 CTD patients). Inter-observer variability was assessed by two radiologists (YT and JW, with 3 and 5 years of experience in CMR imaging, respectively) blinded to all clinical information. Intra-observer variability was measured twice by radiologist YT, with at least 1 month between repeated analysis.

### 2.6 Statistical analysis

Statistical analysis was performed using SPSS version 26.0 (IBM Deutschland GmbH, Armonk, NY, USA) and GraphPad Prism version 10.0.2 (GraphPad Prism Software Inc., San Diego, CA, USA). Continuous variables were summarized as mean ± standard deviation (SD) or median with interquartile range (IQR) and were compared between groups using Student’s *t*-test or Mann–Whitney *U*-test as appropriate based on their normality. Comparisons among three groups were carried out using analysis of variance (ANOVA) with Bonferroni or Games Howell *post hoc* test for normally distributed data or the Kruskal–Wallis test for non-normally distributed data. Categorical variables were presented as frequencies (percentages) and were compared using the Pearson *χ* ^2^ test. Univariable and multivariable linear regression analyses were used to investigate the association of SUA levels with CMR-derived RV and RA strain parameters. Multivariable analyses were adjusted to all variables without collinearity and a *P*-value <0.05 in univariable analysis as well as age, sex, hyperlipidemia, disease duration, pulmonary fibrosis, and PH. Intra-class correlation coefficient (ICC) was calculated to determine the inter- and intra-observer reproducibility of RV and RA strain parameters. A two-sided *P*-value <0.05 was considered statistically significant.

## 3 Results

### 3.1 Patients’ characteristics

The clinical characteristics are summarized in **Table 1**. CTD patients had significantly higher SUA levels [308.5 (249.00, 357.800) vs. 265.30 (222.80, 297.35) umol/L, *P*=0.001] than controls. According to the tertiles of SUA level distribution in the present study, the CTD patients were divided into three subgroups: the first tertile (SUA level <276 umol/L), the second tertile (276–342 umol/L), and the third tertile (>342 umol/L). Approximately, a third of CTD patients had pulmonary involvement and presented in New York Heart Association (NYHA) class III–IV, which had a higher percentage in the third tertile. The median disease duration was 17.50 (5.00, 60.00) months. The CTD patients in the third tertile also had a significantly longer disease duration than those in the first tertile [21.00 (6.00, 66.00) vs. 7.00 (2.00, 52.75) months, *P* = 0.030]. No significant differences were found among age, sex, BMI, BSA, heart rate, systolic blood pressure, and diastolic blood pressure as well as the proportion of hypertension and diabetes mellitus between the CTD patients and the controls (all *P* > 0.05), but the percentage of hyperlipidemia was higher in CTD patients than in the controls (34.6 vs. 9.6%, *P* = 0.001).

**Table 1:** Clinical characteristics of patients with connective tissue disease and controls.

	Controls (*n* = 52)	CTD (*n* = 104)	CTD			CTD with preserved RVEF (*n* = 70)
			First tertile (*n* = 34)	Second tertile (*n* = 36)	Third tertile (*n* = 34)	
Female, *n* (%)	40 (76.9%)	84 (80.8%)	32 (94.1%)	30 (83.3%)	22 (64.7%)^b^	60 (85.7%)
Age, years	46.17 ± 14.02	48.51 ± 12.49	47.47 ± 12.99	48.94 ± 13.25	49.09 ± 11.43	48.80 ± 12.35
BMI, kg/m^2^	22.02 ± 3.43	22.40 ± 3.52	21.97 ± 3.80	22.48 ± 3.69	22.74 ± 3.08	22.52 ± 3.50
BSA, m^2^	1.60 ± 0.15	1.62 ± 0.16	1.59 ± 0.13	1.61 ± 0.16	1.67 ± 0.17	1.61 ± 0.14
Heart rate, beats/min	74.40 ± 15.48	75.22 ± 13.79	73.35 ± 9.63	75.94 ± 16.22	76.32 ± 14.69	75.57 ± 13.41
Systolic blood pressure, mmHg	120.95 ± 16.20	117.96 ± 15.84	116.59 ± 15.44	117.36 ± 15.48	119.97 ± 16.86	119.39 ± 16.54
Diastolic blood pressure, mmHg	76.86 ± 12.69	77.99 ± 12.14	76.97 ± 11.25	79.08 ± 11.28	77.85 ± 14.01	78.60 ± 11.02
Underlying CTD, *n* (%)-						
IIM	–	49 (47.1%)	18 (52.9%)	18 (50.0%)	13 (38.2%)	39 (55.7%)
SLE	–	29 (27.9%)	7 (20.6%)	11 (30.6%)	11 (32.4%)	15 (21.4%)
SSc	–	6 (5.8%)	1 (2.9%)	1 (2.8%)	4 (11.8%)	2 (2.9%)
pSS	–	5 (4.8%)	1 (2.9%)	2 (5.6%)	2 (5.6%)	2 (2.9%)
RA	–	2 (1.9%)	1 (2.9%)	1 (2.9%)	–	1 (1.4%)
MCTD	–	1 (1.0%)	–	1 (2.8%)	–	–
UCTD	–	4 (3.8%)	4 (11.8%)	–	–	4 (5.7%)
Overlap syndrome	–	8 (7.7%)	2 (5.9%)	2 (5.6%)	4 (11.8%)	7 (10.0%)
Disease duration, months	–	17.50 [5.00, 60.00]	7.00 [2.00, 52.75]	24.00 [9.75, 81.00]	21.00 [6.00, 66.00] ^b^	12.00 [3.75, 48.75]
NYHA class III–IV, *n* (%)	–	22 (21.2%)	2 (5.9%)	9 (25.0%)	11 (32.4%)	7 (10.0%)
Pulmonary fibrosis^*^, *n* (%)	–	42 (40.4%)	9 (26.5%)	17 (47.2%)	16 (47.1%)	27 (38.6%)
PH, *n* (%)	–	32 (30.8%)	7 (20.6%)	10 (27.8%)	15 (44.1%)	14 (20.0%)
Pericardial effusion, *n* (%)	–	43 (41.3%)	13 (38.2%)	14 (38.9%)	16 (47.1%)	27 (38.6%)
Hypertension, *n* (%)	7 (13.5%)	21 (20.2%)	5 (14.7%)	11 (30.6%)	5 (14.7%)	17 (24.3%)
Hyperlipidemia^†^, *n* (%)	5 (9.6%)	36 (34.6%)^a^	12 (35.3%)a	13 (36.1%)a	11 (32.4%)^a^	26 (37.1%)^a^
Diabetes mellitus, *n* (%)	5 (9.6%)	6 (5.8%)	3 (8.8%)	1 (2.8%)	2 (5.9%)	4 (5.7%)
SUA, umol/L	265.30 [222.80, 297.35]	308.50 [249.00, 357.80] ^a^	208.00 [190.25, 249.00] ^a^	308.50 [286.25, 326.00] ^ab^	412.15 [357.80, 467.50] ^abc^	282.00 [221.93, 343.50]
eGFR, mL/min/1.73 m^2^	102.00 [90.75, 111.35]	105.45 [87.25, 122.90]	110.60 [90.00, 126.88]	106.45 [95.95, 121.58]	100.30 [79.48, 114.65]	106.60 [90.58, 123.08]
TC, mmol/L	3.99 [3.37, 4.63]	4.27 [3.59, 5.09]	4.42 [3.71, 5.09]	4.31 [3.82, 5.19] ^a^	3.99 [3.41, 5.10]	4.35 [3.78, 5.09]
TG, mmol/L	1.13 [0.83, 1.47]	1.63 [1.03, 2.64] ^a^	1.45 [0.89, 3.00]	1.72 [1.25, 2.36] ^a^	1.79 [1.13, 2.58] ^a^	1.66 [0.98, 2.74] ^a^
HDL-C, mmol/L	1.25 [1.09, 1.43]	1.10 [0.85, 1.32] ^a^	1.14 [0.93, 1.41]	1.12 [0.97, 1.33]	0.95 [0.74, 1.25] ^a^	1.14 [0.94, 1.35]
LDL-C, mmol/L	2.42 [2.01, 3.14]	2.43 [2.01, 3.23] ^a^	2.36 [1.85, 3.02]	2.57 [2.18, 3.31]	2.34 [2.01, 3.35]	2.45 [1.95, 3.34]

Values are presented as the mean ± SD, number (percentage), or median [interquartile range].

CTD, connective tissue disease; BMI, body mass index; BSA, body surface area; IIM, idiopathic inflammatory myopathy; SLE, systemic lupus erythematosus, SSc, systemic sclerosis; pSS, primary Sjogren Syndrome; RA, rheumatoid arthritis; MCTD, mixed connective tissue disease; UCTD, undifferentiated connective tissue disease; NYHA, New York Heart Association; PH, pulmonary hypertension; SUA, serum uric acid; eGFR, estimated glomerular filtration rate; TC, total cholesterol; TG, triglyceride; HDL-C, high-density lipoprotein-cholesterol; LDL-C, low-density lipoprotein-cholesterol; a, *P* < 0.05 versus controls; b, *P* < 0.05 versus patients in the first tertile; c, *P* < 0.05 versus patients in the second tertile.

* As reported on CT thorax imaging by specialist radiologists.

† Total cholesterol ≥ 6.2 mmol/L, triglycerides ≥ 2.3 mmol/L, or low-density lipoprotein ≥ 4.1 mmol/L.

### 3.2 CMR characteristics

**Figure 2:**
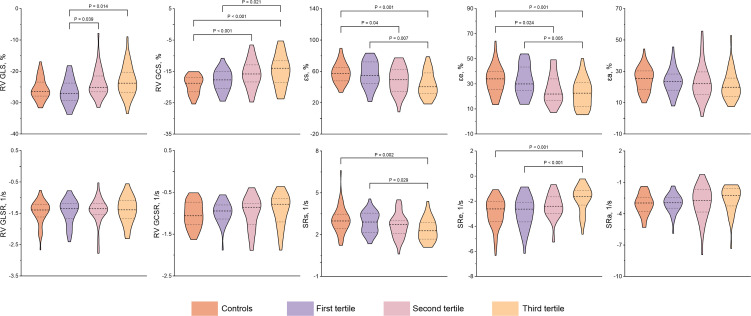
Comparison of RV and RA strain and strain rate between CTD subgroups by the tertiles of SUA and controls. CTD, connective tissue disease; SUA, serum uric acid; RV, right ventricular; GLS, global longitudinal strain; GLSR, global longitudinal strain rate; GCS, global circumferential strain; GCSR, global circumferential strain rate; *ε*_s_, right atrial reservoir strain; *ε*_e_, right atrial conduit strain; *ε*_a_, right atrial booster strain; SRs, reservoir strain rate; SRe, conduit strain rate; SRa, booster strain rate.

**Figure 3:**
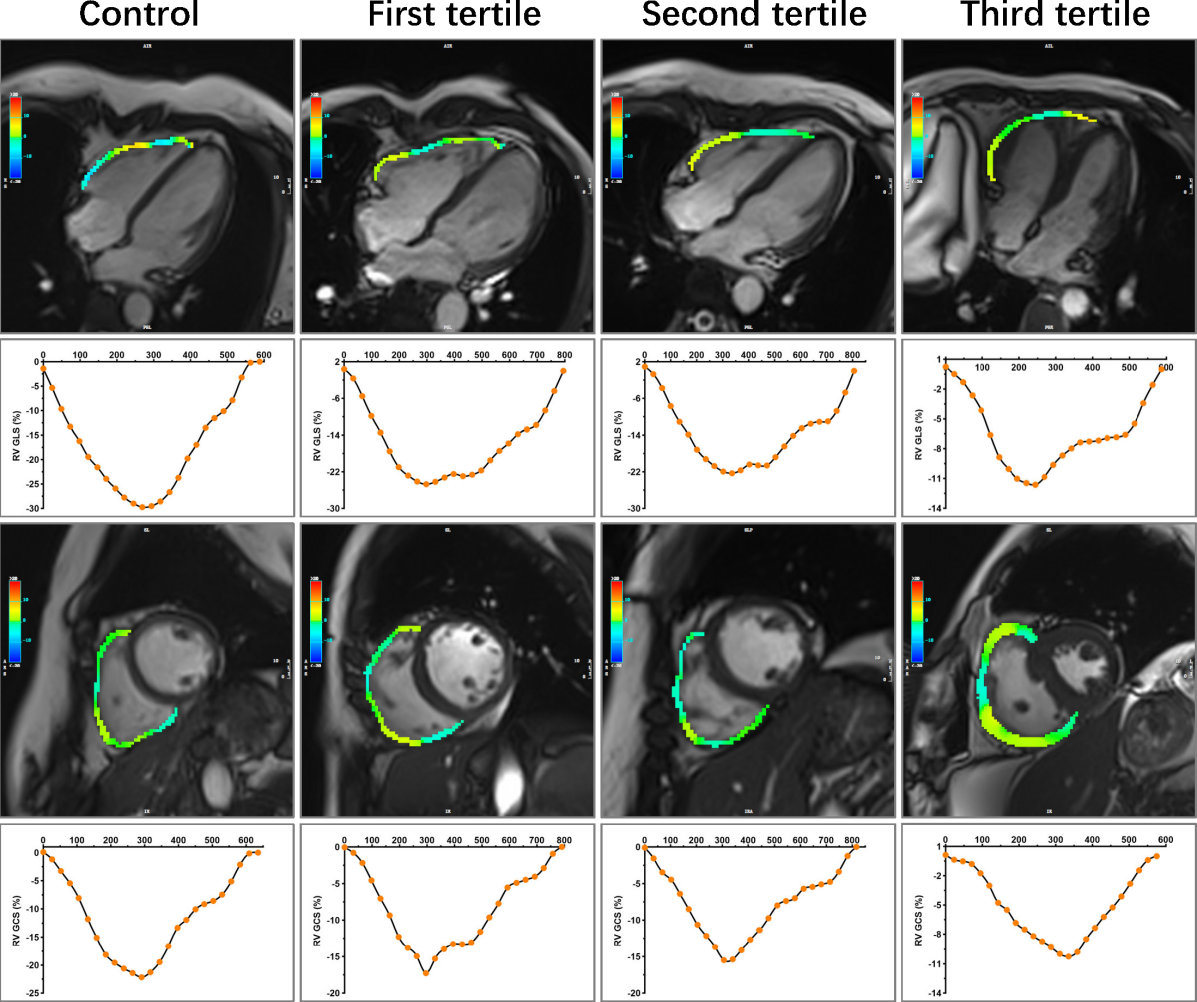
Representative images of RV longitudinal and circumference strain in control and CTD subgroups by the tertiles of SUA. A 47-year-old female served as control. A 50-year-old female with SLE was in the first tertile of SUA, and the disease duration at the time of CMR was 168 months. SUA level = 166 umol/L, sPAP = 51.24 mmHg. A 49-year-old female with SLE was in the second tertile of SUA, and the disease duration at the time of CMR was 120 months. SUA level = 279 umol/L, sPAP = 45.96 mmHg. A 49-year-old female with SLE was in the third tertile of SUA, and the disease duration at the time of CMR was 216 months. SUA level = 427 umol/L, sPAP = 85.00 mmHg. RV, right ventricular; CTD, connective tissue disease; SUA, serum uric acid; SLE, systemic lupus erythematosus; sPAP, systolic pulmonary arterial pressure; GLS, global longitudinal strain; GCS, global circumferential strain.

**Figure 4:**
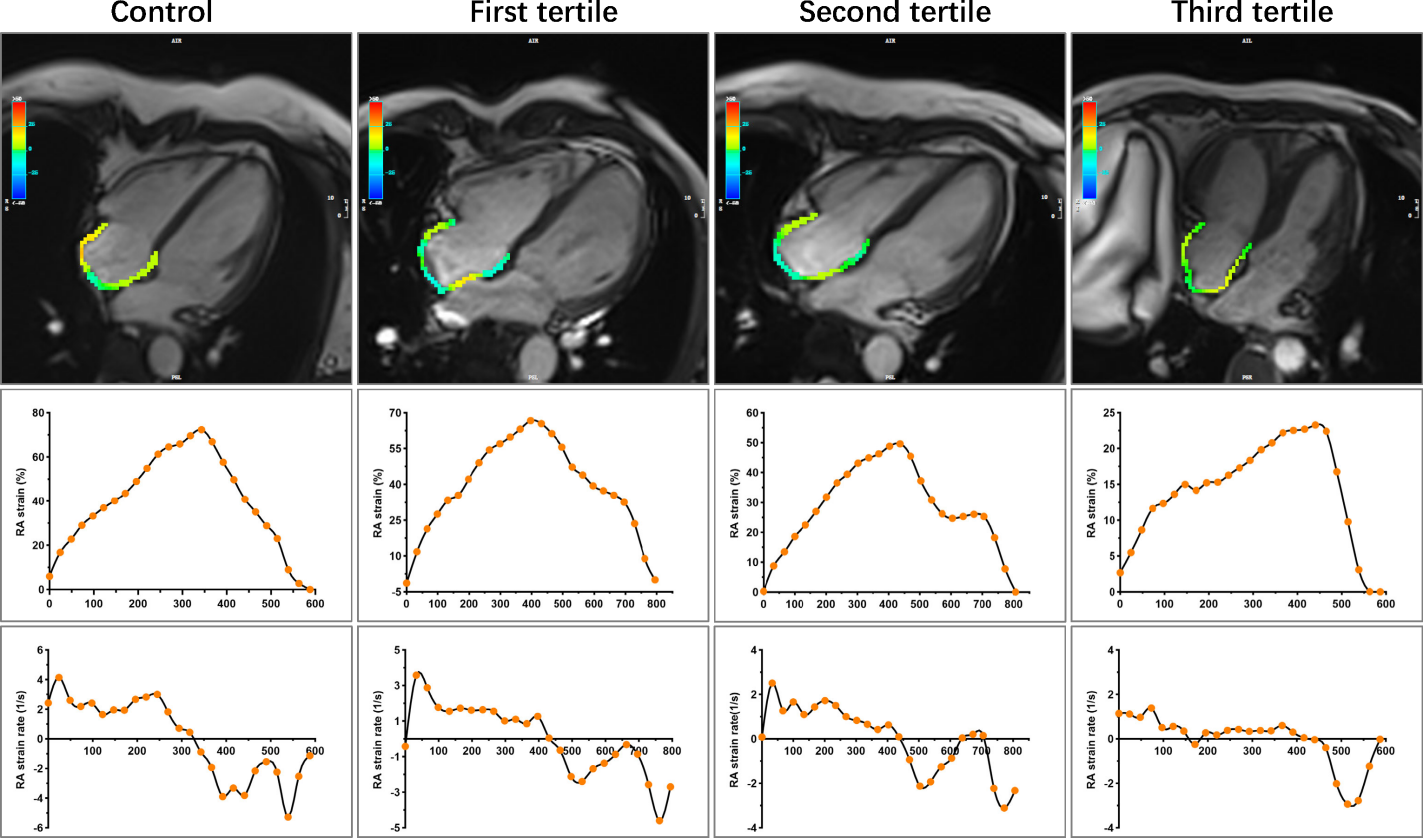
Representative images of RA strain and strain rates in control and CTD subgroups by the tertiles of SUA. Other details are as shown in **Figure 3**. CTD, connective tissue disease; SUA, serum uric acid; RA, right atrial.

The conventional CMR characteristics as well as RV and RA strain parameters of all participants are shown in **Table 2**. The CTD patients had significantly increased RVEDVi, RVESVi, and LV mass index, whereas they have decreased RVEF and RV GCS compared with the controls (all *p* < 0.05), and RV GCS remains impaired even in CTD patients with preserved RVEF (-17.81 ± 3.56 vs. -19.17 ± 2.84%, *P* = 0.025). Although no differences in RA morphological and functional parameters were observed among CTD patients and controls, the CTD patients showed significantly impaired RA strain parameters except SR_a_ [*ε*_s_: 49.52 ± 16.90 vs. 58.04 ± 12.60 %, *P*<0.001; *ε*_e_: 25.92 (17.13, 34.17) vs. 33.95 (25.66, 39.81) %, *P* = 0.002; *ε*_a_: 22.20 (15.73, 27.68) vs. 25.31 (18.39, 30.24) %, *P* = 0.025; SRs: 2.62 ± 0.83 vs. 3.04 ± 0.95 1/s, *P* = 0.005; SR_e_: -2.21 (-3.05, -1.46) vs. -2.62 (-3.62, -2.04) 1/s, *P* = 0.004]. In CTD patients with preserved RVEF, there are significantly increased LV mass index, RVEDVi, RVSVi, RAEF_t_ and RAEF_a_, whereas decreased RAV_min_ index compared with controls (all *P* < 0.05). Both RV and RA strain parameters tended to worsen with increasing SUA levels among subgroups. Compared with patients in the first tertile, those in the third tertile had significantly impaired RV GLS and RV GCS as well as RA reservoir and conduit strain parameters. RV GLS was also significantly lower in the second tertile (all *P* < 0.05). The RV GLSR, RV GCSR, and RA booster strain parameters had no significant difference across tertiles of SUA in CTD patients (all *P* > 0.05). **Figures 2**–**4** show the RV and RA strain parameters in CTD subgroups based on the tertiles of SUA and controls.

**Table 2:** Cardiac magnetic resonance characteristics of patients with connective tissue disease and controls.

	Controls (*n* = 52)	CTD (*n* = 104)	CTD			CTD with preserved RVEF (*n* = 70)
			First tertile (*n* = 34)	Second tertile (*n* = 36)	Third tertile (*n* = 34)	
LV parameters						
LVEF, %	62.97 ± 5.94	62.55 ± 6.40	63.30 ± 5.98	61.29 ± 6.12	63.14 ± 7.06	63.75 ± 5.82
LVEDVi, mL/m^2^	68.51 ± 9.76	71.44 ± 13.36	71.58 ± 11.71	71.64 ± 12.25	71.07 ± 16.15	71.58 ± 12.45
LVESVi, mL/m^2^	25.02 [21.45, 29.55]	25.68 [21.66, 30.27]	24.95 [21.85, 30.11]	27.52 [22.00, 33.64]	24.32 [20.11, 30.15]	24.86 [21.75, 28.53]
LVSVi, mL/m^2^	43.18 ± 7.40	44.18 ± 7.40	45.01 ± 7.54	43.49 ± 7.30	44.07 ± 7.50	45.29 ± 7.40
LV mass index, g/m^2^	38.82 [35.30, 44.75]	44.55 [39.44, 51.05] ^a^	42.57 [38.12, 48.32]	47.46 [39.25, 50.80] ^a^	45.86 [42.92, 58.90] ^a^	45.14 [39.44, 50.31]^a^
RV parameters						
RVEF, %	57.28 ± 5.21	52.64 ± 10.23 a	56.51 ± 5.98	51.30 ± 9.59 ab	50.18 ± 13.02 ^a^	57.95 ± 5.79
RVEDVi, mL/m^2^	62.23 [55.35, 70.40]	70.38 [61.56, 82.89] ^a^	67.47 [61.22, 81.17]	71.71 [62.58, 82.79]	71.78 [62.75, 91.56] ^a^	67.47 [61.22, 76.30] ^a^
RVESVi, mL/m^2^	26.55 [23.87, 30.25]	30.77 [25.73, 40.13] ^a^	28.61 [24.73, 36.30]	30.77 [27.04, 40.46] ^a^	38.47 [24.05, 52.69] ^a^	28.29 [23.88, 34.84]
RVSVi, mL/m^2^	36.68 ± 7.88	37.72 ± 7.88	39.33 ± 7.07	36.56 ± 6.86	37.34 ± 9.48	39.48 ± 7.12 ^a^
RV mass index, g/m^2^	8.86 [8.04, 10.42]	9.87 [7.88, 11.95]	8.67 [7.57, 11.08]	9.37 [7.56, 11.38]	10.93 [8.98, 13.65] ^ab^	8.85 [7.52, 10.73]
GLS, %	-25.67 ± 3.38	-24.42 ± 4.71	-26.49 ± 3.93	-23.12 ± 4.66 ^b^	-23.12 ± 4.90 ^b^	-25.26 ± 3.72
GCS, %	-19.17 ± 2.84	-16.17 ± 4.44 ^a^	-17.91 ± 3.32	-15.79 ± 4.37 ^a^	-14.83 ± 5.03 ^ab^	-17.81 ± 3.56 ^a^
GLSR, 1/s	-1.40 [-1.55, -1.22]	-1.36 [-1.60, -1.17]	-1.35 [-1.67, -1.19]	-1.36 [-1.55, -1.19]	-1.39 [-1.67, -1.10]	-1.37 [-1.53, -1.16]
GCSR, 1/s	-1.04 [-1.27, -0.76]	-0.90 [-1.18, -0.74]	-0.95 [-1.14, -0.80]	-0.86 [-1.27, -0.77]	-0.79 [-1.21, -0.65]	-0.95 [-1.17, -0.71]
RA parameters						
RAEFt, %	57.40 [53.14, 64.34]	60.12 [51.52, 67.18]	61.40 [51.94, 70.88]	61.12 [55.24, 69.32]	55.92 [49.29, 63.55]	61.94 [54.31, 69.37] ^a^
RAEFp, %	26.62 ± 9.67	23.40 ± 11.22	27.31 ± 12.41	23.63 ± 10.49	19.24 ± 9.43 ^ab^	25.53 ± 11.46
RAEFa, %	42.65 ± 10.14	46.33 ± 11.77	45.70 ± 10.89	47.35 ± 13.12	45.89 ± 11.37	47.53 ± 10.73 ^a^
RAV_max_ index, mL/m^2^	31.85 [25.28, 35.91]	30.28 [24.54, 36.99]	28.82 [23.45, 35.80]	32.26 [25.30, 36.24]	32.65 [23.22, 54.12]	28.10 [22.71, 35.59]
RAV_min_ index, mL/m^2^	13.15 [10.01, 15.21]	12.29 [8.41, 16.87]	11.93 [7.86, 14.70]	11.56 [8.79, 14.95]	13.98 [9.03, 18.71]	11.51 [7.93, 13.92] ^a^
RAV_pre-A_ index, mL/m^2^	22.08 [18.17, 27.29]	23.63 [18.09, 29.09]	23.31 [14.61, 27.40]	22.82 [18.31, 28.14]	26.51 [18.72, 31.69]	22.59 [16.39, 25.85]
*ε*_s_, %	58.04 ± 12.60	49.52 ± 16.90 ^a^	56.24 ± 16.15	48.98 ± 16.84 ^a^	43.36 ± 15.62 ^ab^	54.18 ± 16.09
*ε*_e_, %	33.95 [25.66, 39.81]	25.92 [17.13, 34.17] ^a^	29.90 [24.61, 43.41]	21.89 [16.82, 33.97] ^a^	22.57 [11.92, 31.03] ^ab^	29.75 [21.78, 38.27]
*ε*_a_, %	25.31 [18.39, 30.24]	22.20 [15.73, 27.68] ^a^	23.60 [17.90, 28.27]	22.08 [15.05, 29.62]	19.82 [13.86, 25.67]	23.40 [16.46, 29.76]
SRs, 1/s	3.04 ± 0.95	2.62 ± 0.83 ^a^	2.87 ± 0.74	2.65 ± 0.15	2.34 ± 0.80 ab	2.83 ± 0.78
SRe, 1/s	-2.62 [-3.62, -2.04]	-2.21 [-3.05, -1.46] ^a^	-2.63 [-3.65, -2.09]	-2.41 [-2.97, -1.66]	-1.64 [-2.31, -1.15] ^ab^	-2.59 [-3.32, -1.82]
SRa, 1/s	-2.97 [-3.77, -2.42]	-2.70 [-3.53, -1.89]	-2.95 [-3.48, -2.47]	-2.74 [-3.84, -1.68]	-2.24 [-3.25, -1.58]	-2.93 [-3.70, -2.18]

Values are presented as the mean ± standard deviation, number (percentage), or median [interquartile range].

CTD, connective tissue disease; LV, left ventricular; LVEF, left ventricular ejection fraction; LVEDVi, left ventricular end-diastolic volume index; LVESVi, left ventricular end-systolic volume index; LVSVi, left ventricular stroke volume index; RV, right ventricular; RVEF, right ventricular ejection fraction; RVEDVi, right ventricular end-diastolic volume index; RVESVi, right ventricular end-systolic volume index; RVSVi, right ventricular stroke volume index; GLS, global longitudinal strain; GCS, global circumferential strain; GLSR, global longitudinal strain rate; GCSR, global circumferential strain rate; RA, right atrial; RAEF, right atrial emptying fraction; RAV, right atrial volume; ε_s_, right atrial reservoir strain; ε_e_, right atrial conduit strain; ε_a_, right atrial booster strain; SRs, reservoir strain rate; SRe, conduit strain rate; SRa, booster pump strain rate; a, *P* < 0.05 versus controls; b, *P* < 0.05 versus patients in the first tertile; c, *P* < 0.05 versus patients in the second tertile.

### 3.3 Association of SUA and CMR-derived RV and RA strain parameters in CTD patients

In univariable linear regression analyses, the elevated SUA levels were significantly associated with the impaired RV GLS and RV GCS as well as RA reservoir and conduit strain and strain rates (absolute value of *β* = 0.250 to 0.293, all *P* < 0.05). Multivariable linear regression analyses showed that the elevated SUA level was still independently correlated with impaired *ε*_e_ (*β* = -0.212, *P* = 0.035) and RV GCS (*β* = 0.207, *P* = 0.019) in CTD patients after adjustment for age, sex, hyperlipidemia, disease duration, pulmonary fibrosis, and PH as well as excluded the parameters with collinearity (including RVEDVi, RVESVi, RVSVi, RAEF_t_, RAEF_p_, RAEF_a_, RAV_max_ index, and RAV_pre-A_ index). There were no independent associations between SUA levels and right cardiac strain rates (all *P* > 0.05). Other details are presented in **Table 3** and **Supplementary Table S1**.

**Table 3:** Univariable and Multivariable linear regression analyses of serum uric acid and cardiac magnetic resonance-derived parameters on right ventricular (RV) and right atrial (RA) strain parameters in patients with connective tissue disease.

	RV (*β*)				RA (*β*)					
	GLS, %		GCS, %		ε_s,_ %		ε_e_, %		ε_a_, %	
	Univariable	Multivariable	Univariable	Multivariable	Univariable	Multivariable	Univariable	Multivariable	Univariable	Multivariable
SUA, umol/L	0.260^a^	0.127	0.285^a^	0.207^a^	-0.270^a^	-0.163	-0.286^a^	-0.212^a^	-0.107	–
eGFR, mL/min/1.73m^2^	-0.086	–	-0.139	–	0.050	–	0.185		-0.152	–
RVEF, %	-0.629^a^	-0.502^a^	-0.644^a^	-0.665^a^	0.459^a^	0.259^a^	0.463^a^	0.358^a^	0.210^a^	-0.008
RVEDVi, mL/m^2^	0.564^a^	–	0.415^a^	–	-0.343^a^	–	-0.281^a^	–	-0.243^a^	–
RVESVi, mL/m^2^	0.678^a^	–	0.583^a^	–	-0.433^a^	–	-0.407^a^	–	-0.238^a^	–
RVSVi, mL/m^2^	-0.062	–	-0.212^a^	–	0.063	–	0.183	–	-0.127	–
RV mass index, g/m^2^	0.527^a^	0.206	0.355^a^	-0.114	-0.432^a^	-0.182	-0.367^a^	-0.082	-0.289^a^	-0.219
RAEFt, %	-0.384^a^	–	-0.169	–	0.608^a^	–	0.416^a^	–	0.537^a^	–
RAEFp, %	-0.402^a^	–	-0.271^a^	–	0.406^a^	–	0.493^a^	–	0.080	–
RAEFa, %	-0.217^a^	–	-0.033	–	0.485^a^	–	0.190	–	0.613^a^	–
RAV_max_ index, mL/m^2^	0.204^a^	–	0.264^a^	–	-0.238^a^	–	-0.162	–	-0.210^a^	–
RAV_min_ index, mL/m^2^	0.317^a^	0.032	0.224^a^	0.021	-0.493^a^	-0.318^a^	-0.359^a^	-0.190	-0.407^a^	-0.318^a^
RAV_pre-A_ index, mL/m^2^	0.311^a^	–	0.307^a^	–	-0.364^a^	–	-0.325^a^	–	-0.223^a^	–
R^2^		0.501		0.471		0.382		0.297		0.248

Multivariable analyses were adjusted to all variables without collinearity and univariable p-value <0.05 as well as age, sex, hyperlipidemia, disease duration, pulmonary fibrosis, and PH, pulmonary hypertension. Other abbreviations are as shown in **Tables 1** and **2**.

β, value of standardized coefficients.

### 3.4 Reproducibility of RV and RA strain analysis

The inter-observer and intra-observer variability values of the RV and RA strain parameters are presented in **Table 4**. There were excellent intra- and inter-observer agreements in the measurement of RV strain and strain rates (ICC = 0.795 to 0.959 and 0.764 to 0.936, respectively) as well as RA phasic strain and strain rate (ICC = 0.850 to 0.947 and 0.830 to 0.936, respectively).

**Table 4:** Intra- and inter-observer variabilities of cardiac magnetic resonance-derived right ventricular (RV) and right atrial (RA) strain parameters.

	Intra- observer (*n* = 40)		Inter-observer (*n* = 40)	
	ICC	95% CI	ICC	95% CI
RV strain parameters				
GLS, %	0.930	0.872 – 0.962	0.911	0.838 – 0.952
GCS, %	0.959	0.919 – 0.979	0.936	0.882 – 0.965
GLSR, 1/s	0.795	0.645 – 0.886	0.764	0.598 – 0.868
GCSR, 1/s	0.878	0.766 – 0.936	0.807	0.666 – 0.893
RA strain parameters				
*ε*_s_, %	0.933	0.876 – 0.964	0.892	0.805 – 0.941
*ε*_e_, %	0.919	0.853 – 0.956	0.875	0.777 – 0.932
*ε*_a_, %	0.947	0.903 – 0.972	0.936	0.883 – 0.966
SRs, 1/s	0.903	0.822 – 0.948	0.855	0.741 – 0.921
SRe, 1/s	0.850	0.736 – 0.918	0.830	0.702 – 0.906
SRa, 1/s	0.939	0.885 – 0.967	0.917	0.850 – 0.955

Other abbreviations are as shown in **Table 2**.

ICC, intra-class correlation coefficient; CI, confidence interval.

## 4 Discussion

To our knowledge, this is the first study to show the relationship between SUA levels and CMR-FT-derived right atrial and ventricular strain parameters in CTD patients. This study found that CTD patients had significant right cardiac remodeling and higher SUA levels compared to controls, and the SUA levels independently correlated with *ε*_e_ and RV GCS. Therefore, the SUA levels may be a potential risk factor of right cardiac chamber remodeling.

### 4.1 Right cardiac chamber remodeling in patients with CTD

The 2021 European League Against Rheumatism recommendations highlight the importance of cardiovascular risk management in patients with CTD (23). Until now, left heart involvement in patients with CTD has been extensively explored. CTD often combined with a variety of common complications under the influence of systemic proinflammatory state and other factors such as genetic susceptibility, including left heart disease, pulmonary fibrosis, or PH—all of these can affect RV afterload. Adaptive RV remodeling may occur in the early stages of the disease to overcome increased RV afterload and ensure sufficient stroke volume. With the progression of the disease, these compensatory mechanisms gradually become inadequate, leading to RV excessive dilatation, dysfunction, and, eventually, right heart failure (24). Although RVEF typically indicates a poor prognosis, the decreased RVEF will not appear until the late stage of heart failure. CMR-FT has the potential to detect myocardial function abnormalities earlier in the disease. In patients with CTD, there are significant correlations between reduced RVEF and impaired RV GCS and RV GLS. RV GCS remains impaired even in CTD patients with preserved RVEF. These findings were consistent with those of Wu et al. (5). Athletes’ hearts also had a similar RV mechanical pattern in a recent study, with lower RVEF and RV GCS but preserved or even higher RV GLS compared with sedentary controls (25). These changes might be related to modified RV myocardial fiber orientation. Physiologically, RV contraction mainly depends on longitudinal shortening (26). The relative dominance of the circumferentially oriented fibers can be observed when chronically pressure-loaded (27). In patients with PH, the transverse wall motion of the mid-RV is significantly reduced and has stronger relationship with RVEF than longitudinal motion (28). Pettersen et al. (29) also demonstrated a predominant circumferential over longitudinal free wall shortening in patients with a systemic RV.

Furthermore, RV diastolic dysfunction may precede systolic dysfunction in diseases with chronic RV pressure overload. RA, mainly through increased dimension (higher RA reservoir function) and contractility (higher RA booster pump function), maintains RV filling of the stiffened ventricle, whereas passive emptying (conduit function) is the first to decline due to the reduced RA–RV pressure gradient (30, 31). The increased RAEF_t_ and RAEF_a_ and decreased RAV_min_ index may play as a compensatory mechanism in CTD patients with preserved RVEF. RA dilatation indicated the presence of RV diastolic stiffness (32). Excessive RA dilation and stiffness occur as the disease progresses, leading to deterioration of RA phasic functions and atrioventricular uncoupling. In the present study, the RA strain parameters significantly decline in CTD patients without overt RA dilation and dysfunction. Moreover, our findings indicate that *ε*_s_ was positively correlated with RVEF as well as negatively correlated with RAV_min_ index in patients with CTD, consistent with that observed in PH (33, 34). Therefore, RA strain parameters may be more sensitive and earlier predictors of RV overload in patients with CTD.

### 4.2 Association between SUA and right cardiac chamber remodeling

The present results show that patients with CTD had significantly elevated SUA levels compared with the controls, and right cardiac chamber remodeling is more severe in CTD patients with higher SUA level subgroups. However, the precise mechanisms underlying the relationship between elevated SUA and right cardiac chamber remodeling found in this study are still unclear and remain to be elucidated.

The elevated SUA levels may lead to right cardiac chamber remodeling by promoting the increased RV afterload in patients with CTD. The RV is very sensitive to afterload change compared with the left ventricle. In the present study, nearly half of the patients were cases combined with pulmonary involvement, which had a higher percentage in the third tertile. Several studies found that the SUA levels were significantly higher in CTD patients with PH than in those without PH (11–36). Although Castillo-Martínez et al. (10) reported that the baseline SUA levels were similar whether SLE patients had PH or not, the steady hyperuricemia was associated with the development of PH in SLE patients without PH at baseline. In CTD-PAH, the baseline SUA levels also had a positive correlation with pulmonary vascular resistance, and steady hyperuricemia may predict a worsened clinical prognosis (12). The elevated SUA level may promote pulmonary vascular remodeling through increased oxidative stress, depletion of nitric oxide, endothelial dysfunction, vasoconstriction, and proliferation of vascular smooth muscle cells (7). Although Savale et al. (37) did not find the influence of hyperuricemia in pulmonary vascular remodeling, UA metabolism was also disturbed in the remodeled pulmonary vascular walls in PAH. Moreover, Wang et al. (38) observed that the SUA levels were higher in rheumatoid arthritis patients with ILD than in patients without ILD. The SUA levels were positively correlated with KL-6, a biomarker of ILD. They further explored the involvement of UA in the pathogenesis of ILD through *in vitro* cellular experiments and found that UA can induce the epithelial-to-mesenchymal transition in alveolar epithelial cells, which is a critical step in the progression of ILD.

Inflammation may play a key role between elevated uric acid and right heart remodeling. Elevated SUA levels may enhance the inflammatory response and thus affect right cardiac chamber remodeling in patients with CTD. Our data shows that patients in the third tertile had significantly impaired RV GLS and RV GCS as well as RA reservoir and conduit strain parameters compared with those in the first tertile, and RV GLS was also significantly lower in the second tertile—even the first and second tertiles of SUA were in the normal reference range. There was independent association between SUA levels and RV GCS and *ε*_e_ after adjustment for pulmonary fibrosis, PH, RVEF, and other potential confounding factors. Chronic long-term inflammation is one of the main pathological features in patients with CTD and be considered to have a contributing role in cardiovascular involvement as well as is one of the most important causes of elevated SUA levels. Increased SUA can, in turn, exacerbate the inflammatory response, resulting in a vicious cycle (39). Even though SSc-PAH patients have similar pulmonary vascular resistance with idiopathic pulmonary hypertension patients, there was a worse RV function and prognosis in the former (40). The primary myocardial involvement derived by inflammation may be one of the potential causes (41). However, none of these mechanisms is independent. They reinforce and interact with each other, ultimately leading to right cardiac chamber remodeling.

In addition, both SUA levels and right heart remodeling are affected by exercise as a confounding factor. On the one hand, low- and moderate-intensity exercise can improve hyperuricemia (42), but high-intensity exercise may increase uric acid production and reduce excretion, leading to increased SUA (43, 44). On the other hand, long-term and intensive endurance exercise has potential effects on the heart, especially the right side, and even on pulmonary circulation (45). However, only one patient in our cohort has a history of long-term and high-intensity exercise and stopped training in the last 4 years. The effect of exercise was not discussed separately in this study. Nonetheless, previous studies suggested that exercise therapy was beneficial to CTD patients and moderates the side effects of high-dose glucocorticoid treatment (46–48). The influence of exercise on right heart remodeling and finding an appropriate kinesitherapy in CTD patients are worth exploring.

## 5 Limitation

There are some limitations in this study. First, this was a single-center study with a relatively small sample size. This study did not discuss the different types of CTD separately due to the limitations of the sample size, despite which is a heterogeneous group of diseases. Compared with IIM, other CTDs more likely to combine with PH and myocardial involvement may have more obvious right cardiac chamber remodeling and higher SUA levels. However, the common medications used in CTD patients are similar, including glucocorticoids and immunosuppressants, and based on the different symptoms performed personalized treatment. Second, causality cannot be determined due to the cross-sectional design; longitudinal or interventional studies should be conducted to further explore the causal relationship between SUA levels and right heart remodeling. Third, although we observed a correlation between SUA levels and LV mass index similar to previous studies in other diseases (49–51), we did not investigate the correlation further for the reason that to we focused on the right cardiac chamber remodeling in present study. The relationship between SUA levels and myocardial fibrosis, edema, and microvascular perfusion in patients with CTD has also not been investigated in this study due to the same reason. Subsequent studies need to focus on these aspects. Finally, given the lack of follow-up data on patients with CTD, further follow-up studies should be performed to explore the prognostic value of SUA levels and right heart strain parameters.

## 6 Conclusion

In summary, SUA may be a potential risk factor of right cardiac chamber remodeling and is independently associated with impaired *ε*_e_ and RV GCS in patients with CTD, which might be used as a non-invasive and low-cost indicator to determine subclinical right cardiac chamber remodeling.

## Data availability statement

The raw data supporting the conclusions of this article will be made available by the authors, without undue reservation.

## Ethics statement

The studies involving humans were approved by Huazhong University of Science and Technology, Tongji Medical School Affiliated Tongji Hospital Medical Ethics Committee. The studies were conducted in accordance with the local legislation and institutional requirements. The human samples used in this study were acquired from primarily isolated as part of your previous study for which ethical approval was obtained. Written informed consent for participation was not required from the participants or the participants’ legal guardians/next of kin in accordance with the national legislation and institutional requirements. Written informed consent was not obtained from the individual(s) for the publication of any potentially identifiable images or data included in this article because this study protocol was approved by the Institutional Review Board of our hospital (TJ-IRB20230914), and the requirement for written informed consent was waived due to the retrospective design.

## Author contributions

YT: Methodology, Writing – original draft. ZY: Writing – original draft, Writing – review & editing. JW: Methodology, Writing – original draft, Writing – review & editing. DT: Methodology, Writing – review & editing. YL: Methodology, Writing – review & editing. CX: Methodology, Writing – review & editing. LH: Supervision, Writing – review & editing. LX: Supervision, Writing – review & editing.

## References

[1] Vonk MC Vandecasteele E van Dijk AP. Pulmonary hypertension in connective tissue diseases, new evidence and challenges. Eur J Clin Invest. (2021) 51:e13453. doi: 10.1111/eci.13453 33216992 PMC7988614

[2] Mira-Avendano I Abril A Burger CD Dellaripa PF Fischer A Gotway MB. Interstitial lung disease and other pulmonary manifestations in connective tissue diseases. Mayo Clin Proc. (2019) 94:309–25. doi: 10.1016/j.mayocp.2018.09.002 30558827

[3] Xu J Yang W Zhao S Lu M. State-of-the-art myocardial strain by CMR feature tracking: clinical applications and future perspectives. Eur Radiol. (2022) 32:5424–35. doi: 10.1007/s00330-022-08629-2 35201410

[4] Vos JL Butcher SC Fortuni F Galloo X Rodwell L Vonk MC. The prognostic value of right atrial and right ventricular functional parameters in systemic sclerosis. Front Cardiovasc Med. (2022) 9:845359. doi: 10.3389/fcvm.2022.845359 35369297 PMC8969768

[5] Wu R Shi RY An DAL Chen BH Jiang M Bacyinski A. Biventricular tissue tracking demonstrating associations between left ventricular myocardial extracellular volume, pulmonary artery pressure, and reduced right ventricular ejection fraction in patients with systemic lupus erythematosus using cardiovascular MRI. Clin Radiol. (2020) 75:237.e217–237.e225. doi: 10.1016/j.crad.2019.09.136 31679817

[6] Bratis K Lindholm A Hesselstrand R Arheden H Karabela G Stavropoulos E. CMR feature tracking in cardiac asymptomatic systemic sclerosis: Clinical implications. PloS One. (2019) 14:e0221021. doi: 10.1371/journal.pone.0221021 31433819 PMC6703686

[7] Ndrepepa G. Uric acid and cardiovascular disease. Clin Chim Acta. (2018) 484:150–63. doi: 10.1016/j.cca.2018.05.046 29803897

[8] Elera-Fitzcarrald C Reategui-Sokolova C Gamboa-Cardenas RV Medina M Zevallos F Pimentel-Quiroz VR. Serum uric acid is associated with damage in patients with systemic lupus erythematosus. Lupus Sci Med. (2020) 7:e000366. doi: 10.1136/lupus-2019-000366 32153795 PMC7046960

[9] Zou YW Li QH Zhu YY Pan J Gao JW Lin JZ. Prevalence and influence of hypouricemia on cardiovascular diseases in patients with rheumatoid arthritis. Eur J Med Res. (2022) 27:260. doi: 10.1186/s40001-022-00888-5 36411486 PMC9677667

[10] Castillo-Martínez D Marroquín-Fabián E Lozada-Navarro AC Mora-Ramírez M Juárez M Sánchez-Muñoz F. Levels of uric acid may predict the future development of pulmonary hypertension in systemic lupus erythematosus: a seven-year follow-up study. Lupus. (2016) 25:61–6. doi: 10.1177/0961203315600539 26306740

[11] Kim KJ Baek IW Park YJ Yoon CH Kim WU Cho CS. High levels of uric acid in systemic lupus erythematosus is associated with pulmonary hypertension. Int J Rheum Dis. (2015) 18:524–32. doi: 10.1111/1756-185X.12262 24428867

[12] Wang J Wang Y Li X Huang Y Sun X Wang Q. Serum uric acid is associated with disease severity and may predict clinical outcome in patients of pulmonary arterial hypertension secondary to connective tissue disease in Chinese: a single-center retrospective study. BMC Pulm Med. (2020) 20:272. doi: 10.1186/s12890-020-01309-1 33076877 PMC7574226

[13] Deng XL Yi HW Xiao J Zhang XF Zhao J Sun M. Serum uric acid: A risk factor for right ventricular dysfunction and prognosis in heart failure with preserved ejection fraction. Front Endocrinol (Lausanne). (2023) 14:1143458. doi: 10.3389/fendo.2023.1143458 36950688 PMC10025558

[14] Lundberg IE Tjarnlund A Bottai M Werth VP Pilkington C Visser M. 2017 European League Against Rheumatism/American College of Rheumatology classification criteria for adult and juvenile idiopathic inflammatory myopathies and their major subgroups. Ann Rheum Dis. (2017) 76:1955–64. doi: 10.1136/annrheumdis-2017-211468 PMC5736307 29079590

[15] Aringer M Costenbader K Daikh D Brinks R Mosca M Ramsey-Goldman R. European League Against Rheumatism/American College of Rheumatology classification criteria for systemic lupus erythematosus. Ann Rheum Dis. (2019) 78:1151–9. doi: 10.1136/annrheumdis-2018-214819 31383717

[16] van den Hoogen F Khanna D Fransen J Johnson SR Baron M Tyndall A. 2013 classification criteria for systemic sclerosis: an American college of rheumatology/European league against rheumatism collaborative initiative. Ann Rheum Dis. (2013) 72:1747–55. doi: 10.1136/annrheumdis-2013-204424 24092682

[17] Shiboski CH Shiboski SC Seror R Criswell LA Labetoulle M Lietman TM. 2016 American college of rheumatology/European league against rheumatism classification criteria for primary sjogren's syndrome: A consensus and data-Driven methodology involving three international patient cohorts. Arthritis Rheumatol. (2017) 69:35–45. doi: 10.1002/art.39859 27785888 PMC5650478

[18] Villeneuve E Nam J Emery P. 2010 ACR-EULAR classification criteria for rheumatoid arthritis. Rev Bras Reumatol. (2010) 50:481–3. 21125184

[19] Sharp GC Irvin WS Tan EM Gould RG Holman HR. Mixed connective tissue disease–an apparently distinct rheumatic disease syndrome associated with a specific antibody to an extractable nuclear antigen (ENA). Am J Med. (1972) 52:148–59. doi: 10.1016/0002-9343(72)90064-2 4621694

[20] Humbert M Kovacs G Hoeper MM Badagliacca R Berger RMF Brida M. ESC/ERS Guidelines for the diagnosis and treatment of pulmonary hypertension. Eur Respir J. (2022) 44:1312. doi: 10.1183/13993003.00879-2022 36028254

[21] Qu YY Buckert D Ma GS Rasche V. Quantitative assessment of left and right atrial strains using cardiovascular magnetic resonance based tissue tracking. Front Cardiovasc Med. (2021) 8:690240. doi: 10.3389/fcvm.2021.690240 34250043 PMC8264056

[22] Claus P Omar AMS Pedrizzetti G Sengupta PP Nagel E. Tissue tracking technology for assessing cardiac mechanics: principles, normal values, and clinical applications. JACC Cardiovasc Imaging. (2015) 8:1444–60. doi: 10.1016/j.jcmg.2015.11.001 26699113

[23] Drosos GC Vedder D Houben E Boekel L Atzeni F Badreh S. EULAR recommendations for cardiovascular risk management in rheumatic and musculoskeletal diseases, including systemic lupus erythematosus and antiphospholipid syndrome. Ann Rheum Dis. (2022) 81:768–79. doi: 10.1136/annrheumdis-2021-221733 35110331

[24] Vonk-Noordegraaf A Haddad F Chin KM Forfia PR Kawut SM Lumens J. Right heart adaptation to pulmonary arterial hypertension: physiology and pathobiology. J Am Coll Cardiol. (2013) 62:D22–33. doi: 10.1016/j.jacc.2013.10.027 24355638

[25] Fábián A Ujvári A Tokodi M Lakatos BK Kiss O Babity M. Biventricular mechanical pattern of the athlete's heart: comprehensive characterization using three-dimensional echocardiography. Eur J Prev Cardiol. (2022) 29:1594–604. doi: 10.1093/eurjpc/zwac026 35139228

[26] Sanz J Sánchez-Quintana D Bossone E Bogaard HJ Naeije R. Anatomy, function, and dysfunction of the right ventricle: JACC state-of-the-art review. J Am Coll Cardiol. (2019) 73:1463–82. doi: 10.1016/j.jacc.2018.12.076 30922478

[27] Tezuka F Hort W Lange PE Nürnberg JH. Muscle fiber orientation in the development and regression of right ventricular hypertrophy in pigs. Acta pathol japonica. (1990) 40:402–7. doi: 10.1111/j.1440-1827.1990.tb01579.x 2144093

[28] Kind T Mauritz GJ Marcus JT van de Veerdonk M Westerhof N Vonk-Noordegraaf A. Right ventricular ejection fraction is better reflected by transverse rather than longitudinal wall motion in pulmonary hypertension. J Cardiovasc Magn Reson. (2010) 12:35. doi: 10.1186/1532-429X-12-35 20525337 PMC2901360

[29] Pettersen E Helle-Valle T Edvardsen T Lindberg H Smith HJ Smevik B. Contraction pattern of the systemic right ventricle shift from longitudinal to circumferential shortening and absent global ventricular torsion. J Am Coll Cardiol. (2007) 49:2450–6. doi: 10.1016/j.jacc.2007.02.062 17599609

[30] Gaynor SL Maniar HS Prasad SM Steendijk P Moon MR. Reservoir and conduit function of right atrium: impact on right ventricular filling and cardiac output. Am J Physiol Heart Circ Physiol. (2005) 288:H2140–2145. doi: 10.1152/ajpheart.00566.2004 15591102

[31] Gaynor SL Maniar HS Bloch JB Steendijk P Moon MR. Right atrial and ventricular adaptation to chronic right ventricular pressure overload. Circulation. (2005) 112:I212–218. doi: 10.1161/CIRCULATIONAHA.104.517789 16159819

[32] Wessels JN van Wezenbeek J de Rover J Smal R Llucià-Valldeperas A Celant LR. Right atrial adaptation to precapillary pulmonary hypertension: pressure-volume, cardiomyocyte, and histological analysis. J Am Coll Cardiol. (2023) 82:704–17. doi: 10.1016/j.jacc.2023.05.063 37587582

[33] Tello K Dalmer A Vanderpool R Ghofrani HA Naeije R Roller F. Right ventricular function correlates of right atrial strain in pulmonary hypertension: a combined cardiac magnetic resonance and conductance catheter study. Am J Physiol Heart Circ Physiol. (2020) 318:H156–64. doi: 10.1152/ajpheart.00485.2019 31756118

[34] Leng S Dong Y Wu Y Zhao X Ruan W Zhang G. Impaired cardiovascular magnetic resonance-derived rapid semiautomated right atrial longitudinal strain is associated with decompensated hemodynamics in pulmonary arterial hypertension. Circ Cardiovasc Imaging. (2019) 12:e008582. doi: 10.1161/CIRCIMAGING.118.008582 31088152

[35] Chikhoune L Brousseau T Morell-Dubois S Farhat MM Maillard H Ledoult E. Association between routine laboratory parameters and the severity and progression of systemic sclerosis. J Clin Med. (2022) 11:5087. doi: 10.3390/jcm11175087 36079017 PMC9457158

[36] Simpson CE Damico RL Hummers L Khair RM Kolb TM Hassoun PM. Serum uric acid as a marker of disease risk, severity, and survival in systemic sclerosis-related pulmonary arterial hypertension. Pulmon Circ. (2019) 9:2045894019859477. doi: 10.1177/2045894019859477 PMC6664664 31384431

[37] Savale L Akagi S Tu L Cumont A Thuillet R Phan C. Serum and pulmonary uric acid in pulmonary arterial hypertension. Eur Respir J. (2021) 58:2000332. doi: 10.1183/13993003.00332-2020 33446602

[38] Wang Z Wang W Xiang T Gong B Xie J. Serum uric acid as a diagnostic biomarker for rheumatoid arthritis-associated interstitial lung disease. Inflammation. (2022) 45:1800–14. doi: 10.1007/s10753-022-01661-w PMC9197871 35314903

[39] Aghdashi M Behnemoon M Mahmoodi Rad J Rabiepour M. Evaluation of serum uric acid level in systemic lupus erythematosus patients with normal and high pulmonary arterial hypertension. Biomed (Taipei). (2018) 8:16. doi: 10.1051/bmdcn/2018080316 PMC6108231 30141403

[40] Tedford RJ Mudd JO Girgis RE Mathai SC Zaiman AL Housten-Harris T. Right ventricular dysfunction in systemic sclerosis-associated pulmonary arterial hypertension. Circ Heart fail. (2013) 6:953–63. doi: 10.1161/CIRCHEARTFAILURE.112.000008 PMC3815697 23797369

[41] Overbeek MJ Mouchaers KT Niessen HM Hadi AM Kupreishvili K Boonstra A. Characteristics of interstitial fibrosis and inflammatory cell infiltration in right ventricles of systemic sclerosis-associated pulmonary arterial hypertension. Int J Rheumatol. (2010) 2010:604615. doi: 10.1155/2010/604615 20936074 PMC2949592

[42] Hou Y Ma R Gao S Kaudimba KK Yan H Liu T. The effect of low and moderate exercise on hyperuricemia: protocol for a randomized controlled study. Front Endocrinol (Lausanne). (2021) 12:716802. doi: 10.3389/fendo.2021.716802 34539569 PMC8443794

[43] Green HJ Fraser IG. Differential effects of exercise intensity on serum uric acid concentration. Med Sci sports Exercise. (1988) 20:55–9. doi: 10.1249/00005768-198802000-00008 3343917

[44] Huang LL Huang CT Chen ML Mao IF. Effects of profuse sweating induced by exercise on urinary uric acid excretion in a hot environment. Chin J Physiol. (2010) 53:254–61. doi: 10.4077/CJP.2010.AMK060 21793335

[45] Domenech-Ximenos B Garza MS Prat-Gonzalez S Sepulveda-Martinez A Crispi F Perea RJ. Exercise-induced cardio-pulmonary remodelling in endurance athletes: Not only the heart adapts. Eur J Prev Cardiol. (2020) 27:651–9. doi: 10.1177/2047487319868545 31423814

[46] Hu H Xu A Gao C Wang Z Wu X. The effect of physical exercise on rheumatoid arthritis: An overview of systematic reviews and meta-analysis. J Adv Nurs. (2021) 77:506–22. doi: 10.1111/jan.14574 33176012

[47] Dowman LM McDonald CF Hill CJ Lee AL Barker K Boote C. The evidence of benefits of exercise training in interstitial lung disease: a randomised controlled trial. Thorax. (2017) 72:610–9. doi: 10.1136/thoraxjnl-2016-208638 28213592

[48] Nagashima M Takahashi D Mizushima T Yamauchi K. Effects of exercise in patients with connective tissue disease receiving high-dose glucocorticoids: A pilot prospective cohort study. Eur J Appl Physiol. (2021) 121:2253–63. doi: 10.1007/s00421-021-04697-2 33914153

[49] Zhang C Liu R Yuan J Cui J Hu F Yang W. Gender-related differences in the association between serum uric acid and left ventricular mass index in patients with obstructive hypertrophic cardiomyopathy. Biol Sex Differ. (2016) 7:22. doi: 10.1186/s13293-016-0074-x 27054027 PMC4822298

[50] Yoshitomi R Fukui A Nakayama M Ura Y Ikeda H Oniki H. Sex differences in the association between serum uric acid levels and cardiac hypertrophy in patients with chronic kidney disease. Hypertens Res. (2014) 37:246–52. doi: 10.1038/hr.2013.134 24089265

[51] Matsumura K Ohtsubo T Oniki H Oniki H Fujii K Iida M. Gender-related association of serum uric acid and left ventricular hypertrophy in hypertension. Circ J. (2006) 70:885–8. doi: 10.1253/circj.70.885 16799243

