# Supplementary material for: Association of serum uric acid with right cardiac chamber remodeling assessed by cardiovascular magnetic resonance feature tracking in patients with connective tissue disease
Source: Front Endocrinol (Lausanne). 2024 Mar 22;15:1351197. doi: 10.3389/fendo.2024.1351197 (PMC10995324; doi:10.3389/fendo.2024.1351197)
Supplement: Supplementary file 1 [file DataSheet_1.docx]

Supplementary Material

Association of serum uric acid with right cardiac chamber remodeling assessed by cardiovascular magnetic resonance feature tracking in patients with connective tissue disease

# Supplementary Figures and Tables

##
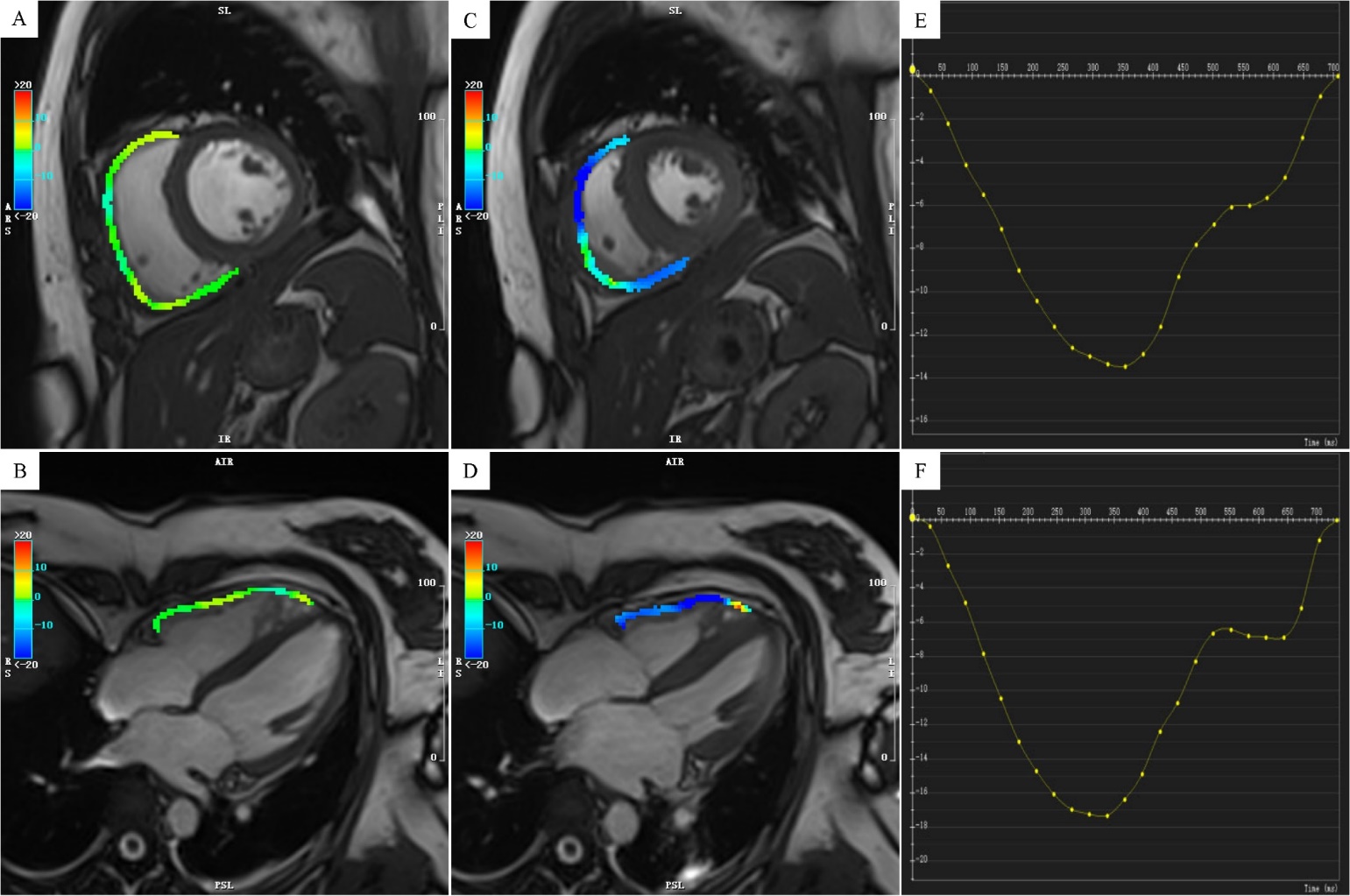
Supplementary Figures

**Supplementary Figure 1.** CMR-FT analysis of RV strains in the standard short-axis and four-chamber long-axis cine images at the end-diastole (A, B) and end-systole (C, D). The end-diastole short-axis and four-chamber long-axis cine images are used to calculate the RV global circumferential (E) and longitudinal (F) strain curve, respectively.
CMR-FT: cardiac magnetic resonance imaging feature tracking; RV, right ventricular.


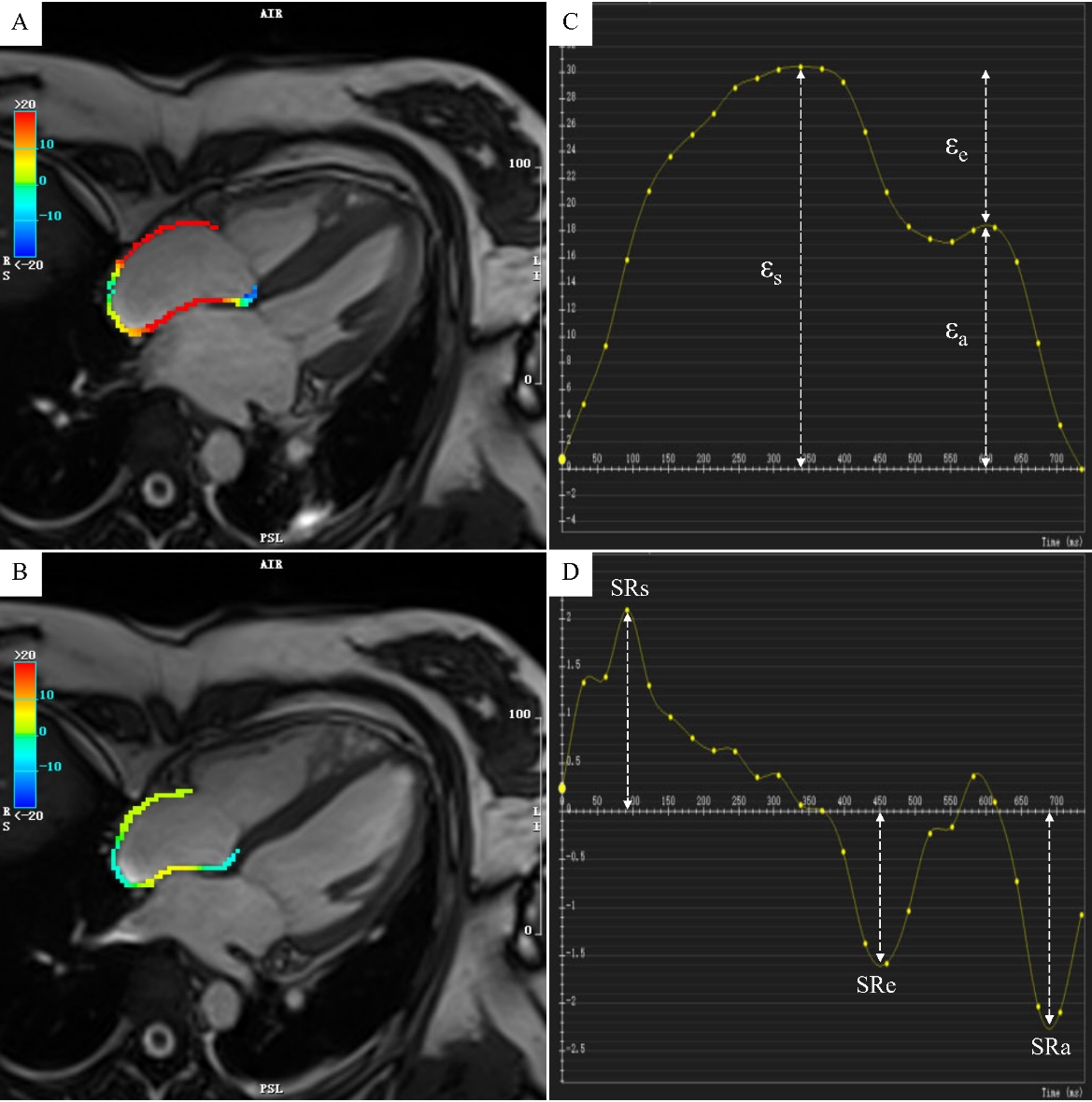


**Supplementary Figure 2**. CMR-FT analysis of RA longitudinal strain parameters in the standard four-chamber long-axis cine images at the end- systole (A) and end- diastole (B). The end-diastolic four-chamber long-axis cine images are used to calculate the RA longitudinal strain (C) and strain rate (D) curves.
CMR-FT, cardiac magnetic resonance imaging feature tracking; RA, right atrial; ε_s_, right atrial reservoir strain; ε_e_, right atrial conduit strain; ε_a_, right atrial booster strain; SRs, reservoir strain rate; SRe, conduit strain rate; SRa, booster strain rate.

## Supplementary Tables

|  | RV (β) | | | | |  | RA (β) | | | | | | | |
| --- | --- | --- | --- | --- | --- | --- | --- | --- | --- | --- | --- | --- | --- | --- |
|  | GLSR, 1/s | |  | GCSR, 1/s | |  | SRs, 1/s | |  | SRe, 1/s | |  | SRa, 1/s | |
|  | Univariable | Multivariable |  | Univariable | Multivariable |  | Univariable | Multivariable |  | Univariable | Multivariable |  | Univariable | Multivariable |
| SUA, umol/L | 0.042 | – |  | 0.088 | – |  | -0.250^*^ | -0.120 |  | 0.293^*^ | 0.176 |  | 0.080 | – |
| eGFR, mL/min/1.73m^2^ | -0.082 | – |  | -0.213^*^ | – |  | 0.033 | – |  | -0.287^*^ | – |  | 0.058 | – |
| RVEF, % | -0.328^*^ | -0.181 |  | -0.540^*^ | -0.499^*^ |  | 0.427^*^ | 0.181 |  | -0.432^*^ | -0.263^*^ |  | -0.225^*^ | 0.027 |
| RVEDVi, mL/m^2^ | 0.461^*^ | – |  | 0.418^*^ | – |  | -0.442^*^ | – |  | 0.305^*^ | - |  | 0.229^*^ | – |
| RVESVi, mL/m^2^ | 0.455^*^ | – |  | 0.522^*^ | – |  | -0.468^*^ | – |  | 0.426^*^ | - |  | 0.241^*^ | – |
| RVSVi, mL/m^2^ | 0.150 | – |  | -0.048 | – |  | -0.073 | – |  | -0.144 | - |  | 0.086 | – |
| RV mass index, g/m^2^ | 0.378^*^ | 0.091 |  | 0.364^*^ | -0.029 |  | -0.440^*^ | -0.145 |  | 0.428^*^ | 0.138 |  | 0.307^*^ | 0.211 |
| RAEFt, % | -0.364^*^ | – |  | -0.357^*^ | – |  | 0.652^*^ | – |  | -0.443^*^ | – |  | -0.583^*^ | – |
| RAEFp, % | -0.320^*^ | – |  | -0.311^*^ | – |  | 0.372^*^ | – |  | -0.595^*^ | – |  | -0.154 | – |
| RAEFa, % | -0.219^*^ | – |  | -0.236^*^ | – |  | 0.566^*^ | – |  | -0.154 | – |  | -0.623^*^ | – |
| RAV_max_ index, mL/m^2^ | 0.289^*^ | – |  | 0.340^*^ | – |  | -0.373^*^ | – |  | 0.233^*^ | – |  | 0.248^*^ | – |
| RAV_min_ index, mL/m^2^ | 0.379^*^ | 0.262^*^ |  | 0.373^*^ | 0.192 |  | -0.593^*^ | -0.456^*^ |  | 0.403^*^ | 0.270^*^ |  | 0.462^*^ | 0.404^*^ |
| RAV_pre-A_ index, mL/m^2^ | 0.368^*^ | – |  | 0.390^*^ | – |  | -0.465^*^ | – |  | 0.417^*^ | – |  | 0.286^*^ | – |
| R^2^ |  | 0.277 |  |  | 0.350 |  |  | 0.480 |  |  | 0.363 |  |  | 0.289 |

Table S1. Univariable and Multivariable linear regression analyses of serum uric acid and CMR-derived parameters on RV and RA strain rates in patients with CTD.

Multivariable analyses were adjusted to all variables without collinearity and univariable P value <0.05 as well as age, sex, hyperlipidemia, disease duration, pulmonary fibrosis, and PH.
CMR: cardiac magnetic resonance; RV, right ventricular; RA, right atrial; CTD, connective tissue disease; β, the value of standardized coefficients, GLSR, global longitudinal strain rate; GCSR, global circumferential strain rate; SRs, reservoir strain rate; SRe, conduit strain rate; SRa, booster pump strain rate; SUA, serum uric acid; eGFR, estimated glomerular filtration rate; RVEF, right ventricular ejection fraction; RVEDVi, right ventricular end-diastolic volume index; RVESVi, right ventricular end-systolic volume index; RVSVi, right ventricular stroke volume index; RAEF, right atrial emptying fraction; RAV, right atrial volume.
^*^P < 0.05
